# Subsurface Transport Behavior of Micro-Nano Bubbles and Potential Applications for Groundwater Remediation

**DOI:** 10.3390/ijerph110100473

**Published:** 2013-12-27

**Authors:** Hengzhen Li, Liming Hu, Dejun Song, Abir Al-Tabbaa

**Affiliations:** 1 Department of Hydraulic Engineering, Tsinghua University, Beijing 100084, China; E-Mail: lihengzhen09@gmail.com; 2 In Situ Solution China Co., Ltd, Nanjing 211106, China; E-Mail: song.dejun@is-solution.com; 3 Department of Engineering, University of Cambridge, Cambridge CB2 1PZ, UK; E-Mail: aa22@cam.ac.uk

**Keywords:** micro-nano bubbles (MNBs), size distribution, hydraulic conductivity, dissolved oxygen (DO)

## Abstract

Micro-nano bubbles (MNBs) are tiny bubbles with diameters on the order of micrometers and nanometers, showing great potential in environmental remediation. However, the application is only in the beginning stages and remains to be intensively studied. In order to explore the possible use of MNBs in groundwater contaminant removal, this study focuses on the transport of MNBs in porous media and dissolution processes. The bubble diameter distribution was obtained under different conditions by a laser particle analyzer. The permeability of MNB water through sand was compared with that of air-free water. Moreover, the mass transfer features of dissolved oxygen in water with MNBs were studied. The results show that the bubble diameter distribution is influenced by the surfactant concentration in the water. The existence of MNBs in pore water has no impact on the hydraulic conductivity of sand. Furthermore, the dissolved oxygen (DO) in water is greatly increased by the MNBs, which will predictably improve the aerobic bioremediation of groundwater. The results are meaningful and instructive in the further study of MNB research and applications in groundwater bioremediation.

## 1. Introduction

Microbubbles and nanobubbles, known as micro-nano bubbles (MNBs) for short, are tiny bubbles with diameters of several tens of micrometers and nanometers, respectively [1,2]. In the past few years, the potential applications of these MNBs in many fields of science and technology have drawn more and more attention, especially in environmental engineering such as surface water remediation because of their special characteristics of large specific surface area, negatively charged surface and high oxygen mass transfer efficiency [2,3,4,5,6,7,8,9,10]. In groundwater remediation, MNBs technology is an advanced form of air sparging which is effective for gasoline remediation [11,12,13,14]. Microbubbles with radii as large as 30 μm were first found to be stable in water and persisted for long periods of time (over 100 h) in contradiction with classical theory by Turner [15]. Nanobubbles were proved to even exist in water for months [7]. One remarkable proven characteristic of MNBs has been that the surface area relates to the pollutant adsorption on the bubble surface [16]. Nanobubbles with a 720 nm diameter are observed to significantly enhance the rate of decomposition of sodium dodecylbenzenesulfonate due to their large surface area that facilitates the reaction [17]. 

MNBs have different swelling/shrinkage properties from macrobubbles. It is reported that the critical diameter separating bubble swelling and shrinkage is about 50–65 μm [18]. Bubbles larger than the critical value will swell, while smaller bubbles will shrink. The coalescence of microbubbles is significantly inhibited by surfactants added into water [19], reducing bubble diameter and affecting bubble numbers [20].

Dissolved oxygen in groundwater is a key ecological factor of the groundwater environment, influencing biological activity and organic matter content [21]. Bioremediation is an important process for the clean-up of contaminated groundwater. The dissolved oxygen content in groundwater plays a significant role in the aerobic biodegradation. Great dissolved oxygen enhancement is expected for MNBs, which will stimulate the microbial activity. The gas dissolution process is related to bubble size, so smaller bubbles should enhance the gas dissolution process [22]. For MNBs, the mass transfer rate of bubble gas from bubbles to surrounding liquid increases with a decrease in the bubble radius and an increase in bubble internal pressure. Oxygen microbubbles stabilized by surfactant, known also as colloidal gas aphrons, can provide higher mass-transfer rates than macrobubbles with millimeter diameters. However, it was qualitatively demonstrated that an increase in the concentration of surfactant in the liquid decreases the mass transfer rate, possibly due to the increased mass-transfer resistance in the gas-water interface [23].

In porous media, emphasis has been put on the zone of influence of traditional air sparging (macrobubbles). The zone of influence has a narrowed scale for macrobubbles which will limit their biodegradation and remediation effects [11,13]. The transport properties of MNBs in porous media are important for the zone of influence of groundwater bioremediation. Whether the MNBs will aggregate and be trapped in the pore water resulting in permeability reduction is basic to study the transport properties. Surfactant-stabilized MNB transport in saturated sand is described as colloid transport [24]. However, the hydraulic conductivities of sand for MNB water with different porosities have not been studied so far. In addition, oxygen transfer efficiency of MNBs has not been intensively investigated. In this study, the MNBs were generated using the spiral liquid flow type method which is more advanced and effective compared to other generation methods [25], yet was not fully studied. The bubble size distribution and its affecting factors were studied using laser-light scattering method. Moreover, the permeability of MNB water through sand was compared with that of air-free water. The oxygen transfer efficiency was quantitatively investigated under different generation conditions and surfactant concentrations. The main objectives of this study were to examine: (1) the transport properties in porous media and (2) the enhanced oxygen transfer efficiency, which was fundamental for further study of MNB applications in groundwater remediation.

## 2. Materials and Methods

### 2.1. Experimental Set-up

#### 2.1.1. Generation Method

**Figure 1:**
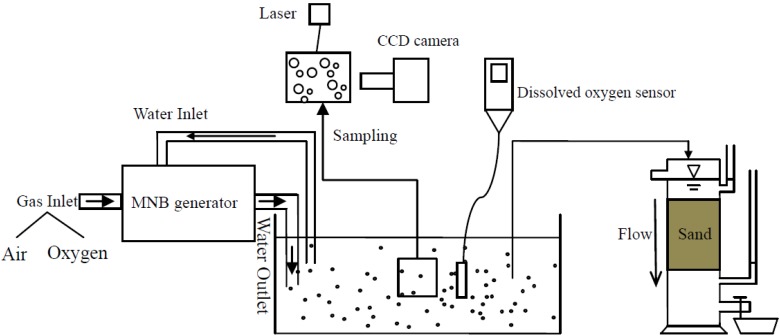
Experimental set-up.

The spiral liquid flow type micro bubble generation method, compared to the Venturi type, ejector type and pressurized dissolution type, has the highest oxygen transfer coefficient, even at a low air flow rate [25]. The gas is injected or absorbed into the cylinder together with the liquid, and then a strong shear force is applied on the liquid to produce a spiral liquid flow, which forms a maelstrom-like cavity in the cylinder. In this way, MNBs are generated in the liquid. The generator used in our study, shown in Figure 1, is a combination of spiral flow and pressurized dissolution, which can produce MNBs with diameters from 400 nm to 100 μm. The generator used in this study has a gas inlet flow rate of 0.24 L/min and water input flow rate of 11 L/min. Thus the MNB water output flow rate is almost 11 L/min. All the tests were operated after 15 min generation of bubbles in a circulation system. 

#### 2.1.2. Bubble Size Distribution Analyzer

The size distribution of MNBs dispersions was measured by the laser-light scattering technique shown in Figure 1, which was feasible for bubble size measurement [26,27]. A Malvern Mastersizer LS13320 (Malvern, Inc., Worcestershire, UK) particle-size analyzer with measuring range from 400 nm to 2,000 μm was employed for this purpose.

#### 2.1.3. Dissolved Oxygen Sensor

The dissolved oxygen in water was measured by the YSI ProODO meter (Yellow Springs, OH, USA) which is an optical, luminescence-based device, shown in Figure 1. The oxygen concentration near the meter sensor was calculated according to the fluorescence quenching theory. Dissolved oxygen ranging from 0 to 50 mg/L (0 to 500% air saturation) with the accuracy of ± 1% (0 to 20 mg/L) and ± 10% (20 to 50 mg/L) could be recorded.

### 2.2. Materials

Experimental materials are listed in Table 1. All the water used in the experiment was deionized water produced by a Direct Q3 system (Merck Millipore Ltd., Billerica, MA, USA). The oxygen cylinder provided 90% concentrated oxygen. The anionic surfactant sodium dodecyl sulfate (SDS) (Beijing Modern Eastern Fine Chemical Co., Ltd., Beijing, China) with the formula CH_3_(CH_2_)_11_OSO_3_Na, was added to water as a surfactant. British standard sand with size ranging from 0.09 mm to 0.15 mm was used for the permeability test with an effective size of 0.11 mm and an average size of 0.14 mm and with a dry density of 1.67 g/cm^3^.

**Table 1:** Materials.

Material	Properties
Water	Deionized water
Gases	Air
	Oxygen (90% concentrated)
Surfactant	Sodium dodecyl sulfate (SDS)
Soil	British standard sand (*D*_10_ = 0.11 mm, *D*_50_ = 0.14 mm)

### 2.3. Experimental Procedure

**Figure 2:**
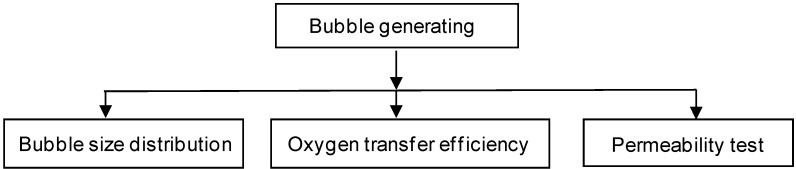
Experimental procedure.

The experimental procedure is shown in Figure 2. The MNB size distribution in water was analyzed under three generation conditions: deionized water with surfactant concentration 0 mg/L, 5 mg/L and 10 mg/L, respectively. After the MNB generation, samples were immediately collected for particle size analysis. The analysis time was shorter than 90 s, which is much less than the long existence time of MNBs observed by the authors and reported in previous research [15], so the bubble size change during the analysis time can be neglected. For each condition, three replicates were done to ensure the precision of the testing results.

In the permeability test, air free water first went through the sand sample to obtain the permeability under saturated conditions, followed by the MNB water going through the same sample. Three soil samples were prepared with the same sand but different porosities which were 0.50, 0.41 and 0.35, respectively. Hydraulic conductivity for each soil sample was obtained by averaging three test results with different hydraulic heads. Then the permeability of MNB water was compared with that of air-free water through sand. The mass transfer features of dissolved oxygen were studied through six different groups of tests, which are shown in Table 2.

**Table 2:** Conditions of oxygen transfer efficiency test.

	Description
Group 1	Air macrobubbles in deionized water
Group 2	Oxygen macrobubbles in deionized water
Group 3	Air micro-nanobubbles in deionized water
Group 4	Oxygen micro-nanobubbles in deionized water
Group 5	Air micro-nanobubbles in deionized water with SDS concentration 5 mg/L
Group 6	Air micro-nanobubbles in deionized water with SDS concentration 10 mg/L

The MNBs generation apparatus started to generate MNBs in a water bucket whose diameter was 25 cm with 11 L water in it for 15 min (the water would be cycled 15 times) at which time the dissolved oxygen (DO) peak value would be reached and then stopped. Meanwhile the DO sensor recorded data until DO became stable. The initial dissolved oxygen value in water was controlled near 0 mg/L using a water vacuum-pumping system. The recording time interval selected was 1 min for the first 30 min and 15 min for the rest time. In the macro bubble experiment, the recording time interval was the same. For each test group, three replicates were done. All the tests mentioned above were carried out at a temperature of 20 °C.

## 3. Results and Discussion

### 3.1. Bubble Size Distribution

**Figure 3:**
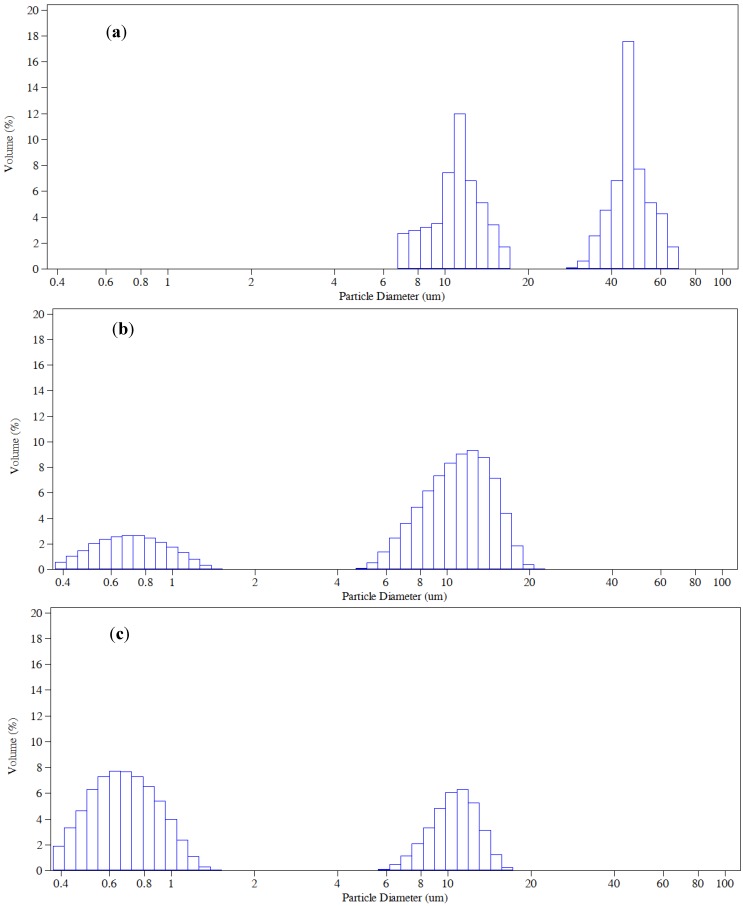
Bubble size distribution in different conditions. (**a**) In deionized water without surfactant. (**b**) In deionized water with surfactant concentration 5 mg/L. (**c**) In deionized water with surfactant concentration 10 mg/L.

The MNBs generated by the spiral liquid flow type apparatus made the water milky-like and it remained so for minutes. The bubble size distribution under different conditions was shown in Figure 3. In all three conditions two size peaks were observed. The average size (*D*_50_), mean bubble size of the first peak (*M*_1P_), and mean bubble size of the second peak (*M*_2P_) are listed in Table 3. In deionized water, the bubble size distribution clustered around 10 μm and 50 μm, which was consistent with Takahashi’s results [28]. Addition of 5 mg/L SDS decreased bubble size with two peaks of 700 nm and 10 μm. 

When the SDS concentration increased to 10 mg/L, the bubble size became slightly smaller with the similar volume percentage peaks but smaller *D*_50_, *M*_1P_ and *M*_2P_. Hence, smaller bubbles were able to form in the surfactant-added water. In the presence of surfactant, the surface tension of gas-water interface was reduced. According to Young-Laplace equation [29,30], the bubble size tended to become smaller to keep the pressure difference. The results were consistent with those found for macro bubbles (diameters of millimeters) by Keitel and Onken [20].

**Table 3:** Bubble sizes under different conditions.

Cases	Deionized Water without Surfactant	Deionized Water with 5 mg/L Surfactant Concentration	Deionized Water with 10 mg/L Surfactant Concentration
D_50_/μm	33 ± 13	9.7 ± 1.3	4.1 ± 1.1
M_1P_/μm	10.7 ± 2.4	0.71 ± 0.16	0.67 ± 0.21
M_2P_/μm	43.0 ± 4.4	10.8 ± 0.8	10.2 ± 1.0

### 3.2. Permeability Results

The porous media used in the tests was fine sand with an average size of 0.14 mm, which was about four times larger than the average bubble size. The mean pore size was calculated by the Kozeny’s equation [31]:

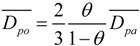
(1)
where *D_po_* is mean pore size, and *D_pa_* is mean particle size and θ is porosity.

The mean pore sizes for soil sample 1–3 were 93.33 μm, 64.86 μm and 50.26 μm, respectively. The hydraulic conductivity of soil samples with air-free water and MNB water was shown in Table 4. When the MNB water goes through the sand, the permeability was slightly lower but no obvious differences from the practical point of view. That is possibly due to two reasons. One is that the MNBs dissolve rapidly into water and become smaller until disappearance. The gas dissolution process is related to bubble size. Smaller bubbles would enhance the gas dissolution process. For MNBs, the mass transfer rate from bubbles to surrounding liquid increases with a decrease in the bubble radius and an increase in bubble internal pressure, which is described in detail in Section 3.3. Therefore, MNBs are less likely to be trapped in sand as a result of the rapid dissolution process. The other reason may be that MNBs adsorption on sand particles is so minimal that the water movement will not be influenced. There may be a maximum adsorption amount of MNBs. And the adsorbed bubbles will not block the water to reduce the permeability. The adsorption of MNBs needs further investigation.

**Table 4:** Test Results for hydraulic conductivity.

	Hydraulic Conductivity/10^−5^ m/s (20 °C)		Ratio
	Air Free Water (k_w_)	Micro-Nano Bubble Water (k_b_)	k_b_/k_w_
Soil sample 1 (porosity = 0.50)	8.1 ± 2.1	7.7 ± 2.3	94%
Soil sample 2 (porosity = 0.41)	6.1 ± 1.3	6.1 ± 2.1	100%
Soil sample 3 (porosity = 0.35)	4.3 ± 1.4	3.8 ± 1.4	88%

In this way, the MNBs transport in porous media (sand) can be described in a solute-like way. It can also be deduced that the MNB water has no influence on the permeability of medium and coarse sand. 

### 3.3. The Oxygen Transfer Efficiency

**Figure 4:**
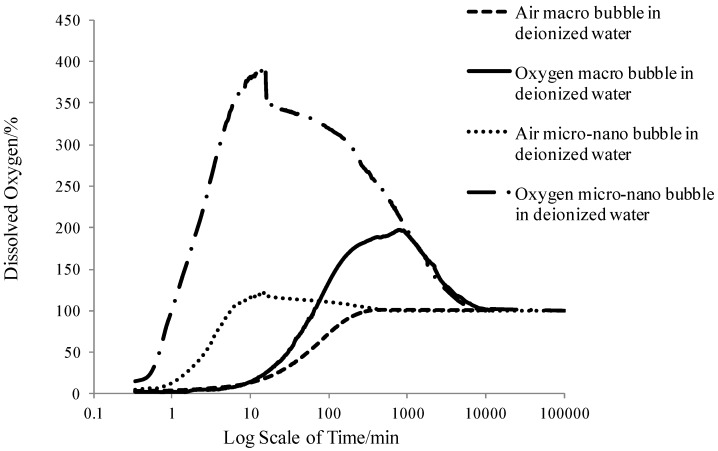
Changing DO pattern with time.

As mentioned above, the spiral liquid flow type MNBs generator applied in this study had better oxygen transfer performance than other types of generators. The DO amount of the first four groups, in the form of percentage over saturation value (9.08 mg/L), changed with time, as shown in Figure 4. Figure 4 shows that MNBs accelerated the oxygen transfer process. For the same gas type, both the increasing DO concentration rate and the peak value of the MNBs were much higher than that of macrobubbles. For the same bubble size, oxygen enhanced the transfer process more than air. According to Fick’s law of diffusion [32], the oxygen transfer efficiency is determined by the oxygen gradient from the inner bubble to the outer liquid. For oxygen MNBs, the oxygen concentration inside the bubble is higher than that inside the air MNBs, and much higher than that inside the air macro bubbles, according to Henry’s law [33] that the gas concentration in liquid is proportional to the gas partial pressure. As shown in Figure 4 the oxygen macrobubbles reached a higher DO peak value than that of air MNBs, it could be seen that a decrease in the bubble size made the oxygen transfer process faster, while the DO peak value was mainly determined by the gas type.

For the air macrobubbles in deionized water test, DO gradually increased with time and finally reached 100%, then remained constant. However, for the tests of MNBs in deionized water, DO increased rapidly and then reached a peak value, which was different for each group, and after that DO went down until it reached 100%. In detail, DO increased rapidly due to the large number of MNBs generated to be dissolved in water. It was nearly a linear increase as shown in Figure 4. The average initial DO increase rate (AIDOIR) was determined, which indicated the mass transfer rate of the MNBs. The AIDOIR relates to the generator capacity of how many, how fast and how tiny bubbles can be generated. The water volume and gas type is associated with AIDOIR as well. Based on Epstein-Plesset model for bubble dissolution in aqueous media, the bubble radius *R* can be written as follows [34]:

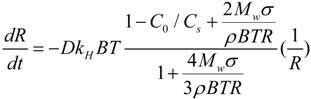
(2)
where *t* is time, *D* is the diffusion coefficient of the gas in the medium, *k*_H_ is the Henry’s law constant, *B* is the gas constant, *T* is temperature, *M*_w_ is the gas molecular weight, σ is surface tension, ρ is the density of gas in the bubble, *C*_0_ is initial gas concentration in solution, *C*_s_ is initial gas concentration in bubble. In this study, *C*_0_ is assumed to be 0. 

For a single bubble, the mass transfer rate *dm/dt* is:

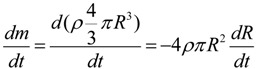
(3)


For test Group 3, an average bubble diameter of 33.4 μm was used and the bubble gas density ρ was calculated using Young-Laplace equation [29,30] and the ideal gas law [35]. 

The internal pressure of MNBs is calculated from the Young-Laplace equation [29,30]:

*P_in_* = *P_out_* + 2*σ* / *R* (4)

where *P*_in_ is the bubble internal pressure, *P*_out_ is the liquid pressure outside the bubble.

Using the ideal gas law [35] we have:

*ρ* = *M_w_P_in_* / *BT* (5)

The density of the gas (ρ) inside bubbles is calculated from Equations (4) and (5):

*ρ* = *M_w_*(*P_out_* + 2*σ* / *R*)/ *BT* 6)

Therefore, for a 33.44 μm-diameter air bubble, the gas density is 1.32 kg/m^3^.

Other parameter values are shown in Table 5 [36]. 

**Table 5:** Simulation parameters.

Parameter	Value	Unit
*D*	1.75 × 10^−9^	m^2^/s
*k_H_*	7.44 × 10^−6^	mol/N·m
*B*	8.3144	J/mol·K
*T*	293.15	K
*M_w_*	29	g/mol
*σ*	0.072	N/m

Therefore, the air mass transfer rate for a single bubble is −5.41 × 10^−7^ mg/min. The bubble number is calculated by the bubble number counter system. The MNB water is placed in the transparent model box with the MNB water irradiated with a laser light, and CCD camera with a microscope can detect the scattered light from the bubbles. In this way, the MNBs can be imaged and recorded in the computer. The bubble number in a certain volume (calculated by the area and depth of image field) is counted. The air MNBs suspension observed is around 7.46 × 10^7^ bubbles in 11 L water. Considering that 21% (volume) of air made up by oxygen, the oxygen mass transfer rate is −9.32 mg/min. Experimentally, the AIDOIR is 0.94 mg/(L·min), so in 11 L water, the total oxygen mass transfer rate is 10.38 mg/min. The simulation result is 10.2% smaller than the experimental results, which proves the experiment is convincing.

AIDOIR relates to the bubble size. Theoretically, an air bubble with a 1 mm diameter weighs approximately eight hundred thousand times the mass of an air bubble with a 10 μm diameter based on the Young-Laplace equation [29,30] and ideal gas law [35]. Similar calculation as above can be carried out for the mass transfer rates of one 1 mm-diameter air bubble and eight hundred thousand 10 μm-diameter air bubbles.

For one 1 mm-diameter air bubble, the mass transfer rate is −3.38 × 10^−6^ mg/min, while for eight hundred thousand 10 μm-diameter air bubbles, the mass transfer rate is −3.68 × 10^−2^ mg/min. Therefore, ten thousand more mass transfer rate will be achieved if one 1 mm-diameter air bubble is divided to eight hundred thousand 10 μm-diameter air bubbles. That explains why the AIDOIR of MNBs is much higher than the macro bubbles in Table 6.

**Table 6:** AIDOIR ^1^, DOPV ^2^ and ST ^3^ of six groups.

	AIDOIR (10^−2^ mg/L/min)	DOPV (mg/L)	ST (10^3^ min)
Group 1 ^1^	2.70 ± 0.20	9.89 ± 0.08	-
Group 2 ^4^	3.50 ± 0.30	19.1 ± 1.2	9.30 ± 0.12
Group 3 ^4^	94.4 ± 2.1	10.4 ± 0.3	0.58 ± 0.06
Group 4 ^4^	342 ± 19	34.2 ± 1.9	9.98 ± 0.14
Group 5 ^4^	74.1 ± 0.5	9.63 ± 0.07	1.86 ± 0.08
Group 6 ^4^	63.8 ± 0.3	9.57 ± 0.05	2.58 ± 0.09

^1^ Average initial dissolved oxygen increasing rate; ^2^ Dissolved oxygen peak value; ^3^ Stagnation time; ^4^ Table 2.

As DO concentration increased, the DO concentration gradient between the water and the surrounding environment increased resulting in the DO diffusion from water to the atmosphere. The diffusion rate was proportional to the concentration gradient according to the Fick’s law. At a certain time, the rate of increasing DO concentration was equal to the diffusion rate, and the DO kept stable. In the tests of this study, the time was 15 min, which depended mainly on the generator capacity and water volume. When DO was stable, the DO value was defined as the DO peak value (DOPV). DOPV is the integral result of AIDOIR over a certain time (15 min). Therefore, it is determined by AIDOIR.

When DOPV was reached, the generator was stopped, so the DO is decreasing due to the DO diffusion from the water to the atmosphere. The time when DO decreased from DOPV to a stable value (100%) was defined as stagnation time (ST). ST indicated the life span of MNBs (the time all MNBs dissolve into water) and the diffusion rate. It was associated with the MNB properties: size, number, interface properties and gas type. Water volume and diffusion rate also influenced ST.

The diffusion rate was related to the DOPV. Higher DOPV led to higher diffusion rate. However, more DO diffused for higher DOPV. Therefore, ST was a complicated parameter which is needed further study. In this study, ST was a parameter to roughly show the durability of DO increasing effect.

Those three parameters (AIDOIR, DOPV and ST) of all six groups were listed in Table 6. The AIDOIR of the oxygen micro-nanobubble group shown in Table 6 was nearly 125 times faster than the air macrobubble group and the DOPV was nearly three times larger. Moreover, the air micro-nanobubble group (Group 3) achieved about 35 times the AIDOIR and 1.05 times the DOPV of the air macrobubble group (Group 1). Obvious enhancement in oxygen transfer efficiency could be seen from the comparison. With the surfactant added into the water, as shown in Groups 5 and 6, the AIDOIR reduced and was inversely proportional to the surfactant concentration compared to Group 3. The same trend was also found in the DOPV analysis. The surfactant concentration influenced the bubble interface properties relating to the oxygen transfer process. Higher surfactant concentration would exert larger mass transfer resistance on the interface.

The gas type played a major role in the parameter ST, that is to say oxygen bubbles, regardless of sizes, had a greater durability performance than air bubbles (at least four times longer). Groups 3, 5 and 6 indicated that surfactant extended the stagnation time probably because the surfactant slowed down the bubble dissolving process. 

From the results above, it was clear that the surfactant has an impact on the gas-water interface properties which would not only influence the bubble formation process, as mentioned in Section 3.1, but also the physical behavior. In a word, higher surfactant concentration would make the bubble size smaller, the oxygen transfer efficiency lower and the stagnation time of MNBs in water longer. Therefore, whether the surfactant needs to be added into water for the MNBs formation has to be carefully taken into consideration.

## 4. Conclusions

Physical properties and transport behavior in a porous medium of MNBs was studied and evaluated in this paper. The main conclusions are as follows:

(1) Two peaks were found in the size distribution of MNBs generated by the spiral liquid flow type generator, one was 10 μm and the other was 50 μm. Adding surfactant into water made the bubble size smaller, with two peaks of 700 nm and 10 μm. Higher surfactant concentration led to smaller bubble size;
(2) MNB water had no influence on the hydraulic conductivity of fine sand;
(3) MNBs greatly enhanced the oxygen transfer efficiency compared to the macro bubbles, which would predictably facilitate the aerobic biodegradation process for groundwater contaminant removal. Oxygen MNBs had the fastest DO mass transfer rate which was nearly 125 times faster than air macro bubble, the highest DO peak value which was nearly three times larger than air macro bubble, the longest dissolved oxygen enhancement durability which was 16 times longer than air micro-nanobubbles. Higher surfactant concentration exerted larger mass transfer resistance on the bubble interface resulting in lower oxygen transfer efficiency and longer stagnation time of MNBs in water.

Based on the results, it is proposed that the MNBs technology is a promising, environmentally friendly technique for groundwater remediation. MNB water has high dissolved-oxygen content and mass transfer efficiency, and can flow in subsurface without change of the hydraulic conductivity. MNBs have long stagnation times and migrate as a colloid in groundwater with a large zone of influence due to the dispersion effect. This paper presented a preliminary study on potential application of MNBs for groundwater remediation, and the physical properties of the MNBs, especially in porous media, are yet to be explored further.
